# Age-related changes in energy metabolism in peripheral mononuclear blood cells (PBMCs) and the brains of cognitively healthy seniors

**DOI:** 10.1007/s11357-023-00810-9

**Published:** 2023-06-13

**Authors:** Carmina V. Silaidos, Martina Reutzel, Lena Wachter, Fabian Dieter, Nasir Ludin, Werner F. Blum, Stefan A. Wudy, Silke Matura, Ulrich Pilatus, Elke Hattingen, Johannes Pantel, Gunter P. Eckert

**Affiliations:** 1 Laboratory for Nutrition in Prevention and Therapy, Biomedical Research Center Seltersberg (BFS), Institute of Nutritional Sciences, Justus-Liebig-University of Giessen, Schubertstrasse 81, 35392 Giessen, Germany; 2 Institute for Neuroradiology, University Hospital, Goethe University, Schleusenweg 2-16, Frankfurt, Germany; 3 Laboratory for Translational Hormone Analytics in Pediatric Endocrinology, Peptide Hormone Research Unit Division of Pediatric Endocrinology and Diabetology, Center of Child and Adolescent Medicine, Justus Liebig University, Giessen, Germany; 4 Department of Psychiatry, Psychosomatic Medicine and Psychotherapy, University Hospital, Goethe University, Frankfurt, Germany; 5 Brain Imaging Center (BIC), University Hospital Frankfurt, Frankfurt a. M, Germany; 6 Geriatric Medicine, Institute of General Practice, Goethe University, Frankfurt a. M, Germany

**Keywords:** Mitochondrial dysfunction, Aging, Peripheral blood mononuclear cells, PBMCs, ^1^H-magnetic resonance spectroscopic imaging, IGF-1, Brain aging, ^31^P magnetic resonance spectroscopic imaging, Adenosine triphosphate, Creatine, Phosphocreatine, SOD, CAD, TFAM, Citrate synthase, Mitochondria, Respiration

## Abstract

Mitochondrial dysfunction is a hallmark of cellular senescence and many age-related neurodegenerative diseases. We therefore investigated the relationship between mitochondrial function in peripheral blood cells and cerebral energy metabolites in young and older sex-matched, physically and mentally healthy volunteers. Cross-sectional observational study involving 65 young (26.0 ± 0.49 years) and 65 older (71.7 ± 0.71 years) women and men recruited. Cognitive health was evaluated using established psychometric methods (MMSE, CERAD). Blood samples were collected and analyzed, and fresh peripheral blood mononuclear cells (PBMCs) were isolated. Mitochondrial respiratory complex activity was measured using a Clarke electrode. Adenosine triphosphate (ATP) and citrate synthase activity (CS) were determined by bioluminescence and photometrically. N-aspartyl-aspartate (tNAA), ATP, creatine (Cr), and phosphocreatine (PCr) were quantified in brains using ^1^H- and ^31^P-magnetic resonance spectroscopic imaging (MRSI). Levels of insulin-like growth factor 1 (IGF-1) were determined using a radio-immune assay (RIA). Complex IV activity (CIV) (− 15%) and ATP levels (− 11%) were reduced in PBMCs isolated from older participants. Serum levels of IGF-1 were significantly reduced (− 34%) in older participants. Genes involved in mitochondrial activity, antioxidant mechanisms, and autophagy were unaffected by age. tNAA levels were reduced (− 5%), Cr (+ 11%), and PCr (+ 14%) levels were increased, and ATP levels were unchanged in the brains of older participants. Markers of energy metabolism in blood cells did not significantly correlate with energy metabolites in the brain. Age-related bioenergetic changes were detected in peripheral blood cells and the brains of healthy older people. However, mitochondrial function in peripheral blood cells does not reflect energy related metabolites in the brain. While ATP levels in PBMCs may be be a valid marker for age-related mitochondrial dysfunction in humans, cerebral ATP remained constant.

**Supplementary Information:**

The online version contains supplementary material available at 10.1007/s11357-023-00810-9.

## Introduction

Healthy mitochondrial function is essential to maintain cellular energy metabolism. This is especially relevant in tissues that consume considerable quantities of energy, such as the brain. Mitochondrial dysfunction induces cellular senescence and many other age-related neurodegenerative diseases [1-5]. For the study of brain aging processes and their possible transition to pathological states, it is of great interest whether mitochondrial function in peripheral cells reflects conditions in the brain. If so, it would be an easily accessible surrogate parameter to assess the energetic situation in the brain and might pave the way to introducing peripheral mitochondrial function as a surrogate parameter in therapeutic or preventive studies investigating pathological conditions associated with energy metabolism. Studies of skeletal muscle tissue are considered as the gold standard for determining mitochondrial parameters in peripheral human tissue [6-8]. More recently, however, studies of peripheral blood mononuclear cells (PBMCs) have become the focus of interest [9, 10], as they are particularly well suited for taking blood-based bioenergetic measurements. As well as playing an important role in immune response and metabolism [11], PBMCs share much of the non-synaptic biochemical environment of neurons. In addition, all epigenetic enzymes and machinery are present in both cell types [12]. In a previous study using PBMCs, we investigated whether a correlation exists between cerebral and peripheral markers of cerebral energy metabolism as measured in vivo in healthy young subjects and we applied MR spectroscopic imaging (MRSI), which provides a non-invasive method to measure brain metabolites [13]. We could demonstrate no significant correlation, which was probably because the number of subjects in the pilot study was too low, and sex differences in the parameters of interest was too high. However, the data showed a tendency towards such a correlation, indicating that the power of our study was insufficient [9]. For this reason, we decided to test for the suspected correlation in a larger sample. Since the previous study revealed significant differences in the measured parameters between males and females, care was taken to balance the two sexes [9]. As mitochondrial dysfunction contributes significantly to aging processes and is a common final pathway in brain aging and dementia-related diseases, we also included a group of older women and men in good mental health. Our study population thus allowed us to disentangle the influence of age and sex on both, cerebral and peripheral markers of energy metabolism. Another novelty in our study was the identification of phosphorus-energy metabolites in the brain, as this allowed a direct comparison between cerebral and peripheral adenosine triphosphate (ATP) levels. Using a cross-sectional observational study involving 65 young and 65 older sex-matched volunteers in good physical and mental health, we sought to test the following hypotheses:

1. Differences in energy metabolism can be detected in both PBMCs and brains of cognitively healthy seniors when compared to the same markers of energy metabolism in healthy young people.
2. When measured in-vivo, markers of energy metabolism in blood cells correlate with energy metabolites in the brain.
3. Sex differences can be observed across age groups in all measured mitochondrial parameters.

## Material and methods

### Study design and participants

**Fig. 1:**
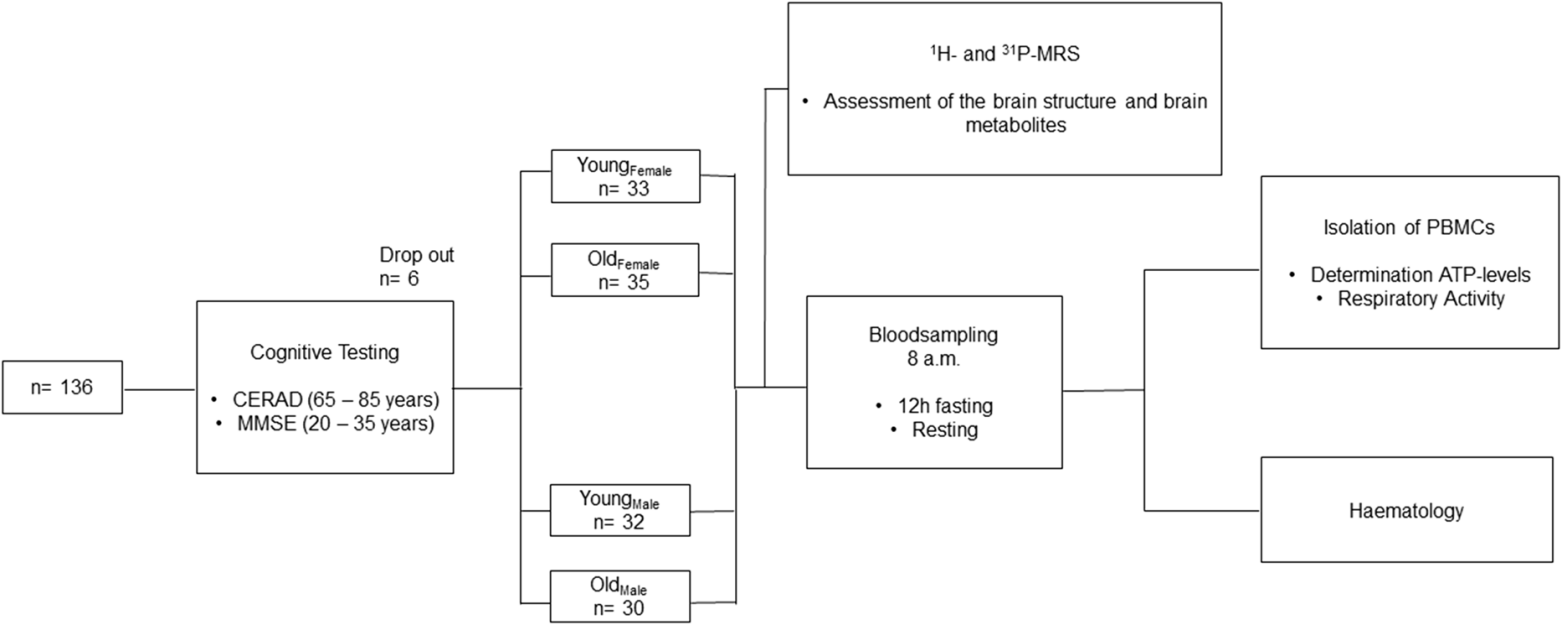
Study design

The Ethics Committee of the Medical Faculty of Goethe University Frankfurt in Germany (reference no. 45/16) approved the study, which we performed in accordance with the Declaration of Helsinki (Version Fortaleza 2012). Taking into account the in- and ex-clusion criteria (Table 1), 136 male and female volunteers were recruited, with most coming from the community of students and employees, the University of the Third Age of Goethe University Frankfurt, and the Lions Club Usingen-Saalburg (Germany). All subjects declared that they understood the experimental procedure and provided their written informed consent. Age, weight, medical history, current medication, smoker status, and blood parameters were recorded (Table 2). Exclusion criteria for participation were pacemaker implants, neurostimulators and drug pumps, metal in the body, claustrophobia, stroke, dementia, a known iron deficiency, pregnancy or lactation, hemophilia, and hematophobia (Table 1). Existing dementia or mild cognitive impairment (MCI) was another exclusion criteria, as neurodegenerative diseases and other chronic brain disorders causes the measured parameters to develop differently from those in healthy subjects [14, 15]. Study participants were therefore asked about pre-existing mild cognitive impairment (MCI) and/or a diagnosis of dementia. To further ensure cognitive health, criteria of major or minor neurocognitive disorder (e.g., dementia or mild cognitive impairment) were applied according to the definition of DSM 5 [16]. Therefore participants were cognitively tested by study personnel trained in neuropsychology. The Mini-Mental State Examination (MMSE) [17] was used to test all participants. In addition, the more extensive Consortium to Establish a Registry for Alzheimer’s Disease (CERAD) test battery and a 10-word recall test were used in the elderly participants [16-18] (Fig. 1). The threshold for cognitive impairment was set at an MMSE score ≤ 27 and/or performance 1.5 standard deviations below normal in each cognitive domain of the CERAD test battery. Following these exclusion criteria none of the participants had to be excluded. Six volunteers discontinued participation because of circulatory problems or illness, leaving 130 subjects to take part in the study (Fig. 1). Overall, 65 healthy young volunteers (33 females and 32 males, mean age 26.6 ± 3.9 years) and 65 healthy elderly volunteers (35 females and 30 males, mean age 71.7 ± 5.7 years) were included (Table 2). All participants underwent magnetic resonance spectroscopy (MRS) at the Brain Imaging Center (BIC) Frankfurt, Germany, for the assessment of brain structure and metabolites. Blood samples were collected in EDTA/K_2_-coated Sarstedt Monovettes (#02.1333.001) from vena brachialis. PBMCs were isolated and investigated at the Institute for Pharmacology, Biocenter Riedberg, Goethe University Frankfurt, and the Institute of Nutritional Sciences, Biomedical Research Center, at the Justus Liebig University of Giessen.

**Table 1:** Inclusion and exclusion criteria

Inclusion criteria	Exclusion criteria
Age-range: 20 to 35 years or 65 to 85 years	Blood parameters
Willingness and ability to consent	- Hemophilia
	- Hematophobia
	- Taking anticoagulants - Mild or major neurocognitive disorder (e.g., dementia or mild cognitive impairment according to DSM 5)
	MRI - Pacemaker - Neurostimulator or drug pump - Metal in the body (e.g., splinters, clips or staples after surgery, but also metal splinters in the body, e.g., from working in the metal processing industry) - Fear of confined spaces (claustrophobia)

**Table 2:**
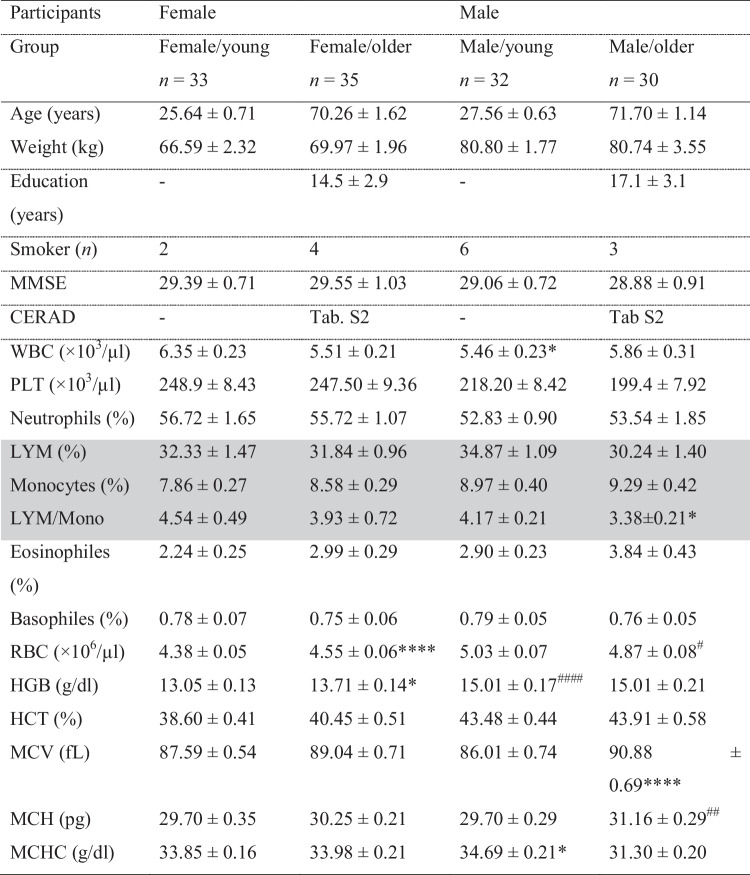
Demographic and blood data

*ns* not significant, *WBC* leucocytes, *PLT* thrombocytes, *LYM* lymphocytes, *RBC* erythrocytes, *HGB* hemoglobin, *HCT* hematocrit, *MCV* mean erythrocyte individual volume, *MCH* mean corpuscular hemoglobin, *MCHC* mean corpuscular hemoglobin concentration

Values are represented as means ± SEM; one-way ANOVA with *p < 0.05, **p < 0.01, ***p < 0.001 and ****p < 0.0001 compared to young controls of the same gender, # indicates differences between sexes (between young female and male; or older female and male with ^#^*p* < 0.05, ^##^*p* < 0.01, ^###^*p* < 0.001. The parameters related to PBMCs are highlighted in gray

Overall, 136 people were recruited for the study. The Mini-Mental State Exam (MMSE) and the more extensive Consortium to Establish a Registry for Alzheimer’s Disease (CERAD) test battery were used to test the cognitive status of participants. No participant had to be excluded because of cognitive dysfunction. Six volunteers withdrew from study participation because of health issues, so that a total of 130 test persons were examined. Blood was taken from the cohort at 8 a.m. All participants underwent proton and phosphorous magnetic resonance spectroscopy (^1^H- and ^31^P-MRS) of the brain to assess structure and levels of cerebral energy metabolites. Blood samples were taken to isolate the peripheral blood mononuclear cells (PBMCs), with density gradient centrifugation being used to measure mitochondrial activity and adenosine triphosphate (ATP) levels. Hematological parameters were assessed in whole blood samples.

### Blood analysis

The differential blood count and all other blood tests were performed by a specialized external laboratory (Laborarztpraxis, Frankfurt, Germany) under GLP conditions and according to standardized procedures.

### Isolation of peripheral blood mononuclear cells (PBMCs)

After overnight fasting and before MRS, blood was taken from each participant in EDTA/K2-coated Sarstedt Monovettes and stored on ice until isolation. One to two hours later, PBMCs were isolated at room temperature from the peripheral blood by diluting it 1:1 with phosphate buffered saline (PBS) and carefully pouring it onto the porous barrier of Leucosep tubes (Greiner Bio-One, Frickenhausen, Germany). Beforehand, the Leucosep tubes had been filled with Biocoll separating solution and centrifuged at 1000 g for 30 s, so that the separating solution was below the barrier. The tubes were centrifuged at 1000 g for 10 min (acceleration (ACC) = 6; deceleration (DEC) = 0). Isolated PBMCs were collected and washed with PBS (25 ml) and centrifuged at 100 g for 10 min (ACC = 9; DEC = 6). The pellet was resuspended in 25-ml PBS and again centrifuged at 100 g for 10 min (ACC = 9; DEC = 6). ATP levels were measured directly after PBMC isolation, the pellet was resuspended in 1-ml RPMI medium (11.1-mM glucose, supplemented with 3% FBS, 50 units/ml penicillin, 50 units/ml streptomycin; 261,870), and for oxygen consumption in 1-ml Mir05 medium (developed by Oroboros, Innsbruck, Austria). Cells were counted using a cell counter (BioRad, Munich, Germany).

### FACS analysis of PBMCs

Flow cytometry as well as its evaluation was performed in the FACS Core Facility of Giessen University Hospital. A cell viability assay was done by staining the dead cells with Cytox Pacific Blue. Composition of the PBMC samples was determined by detecting antibodies. Antibodies against the following clusters of differentiation (CD) were identified: CD56 (NK cells), CD14 (myelo-monocytic cells), CD16 (neutrophils, NK cells, macrophages), CD3 (T cells), and CD19 (B cells) (Tab S1).

### Determination of ATP levels in PBMCs

The PBMCs were cultured in RPMI medium 96-well plates (1 × 10^4^ cells/100 µl/per well) and incubated at 37 °C in an atmosphere containing 5% CO_2_ for 3 h. ATP concentrations were determined using the ViaLight® Plus bioluminescence kit (Lonza, Walkersville, USA), as previously described [9]. The test combines ATP and luciferin in the presence of luciferase to produce light.

### Oxygen consumption in permeabilized PBMCs

Mitochondrial respiration was investigated using the Oxygraph-2 k system (Oroboros, Innsbruck, Austria) and analyzed using DatLab version 7.0.0.2 software. A protocol by Prof. Dr. Erich Gnaiger (University of Innsbruck, Austria) was used to separately measure oxygen consumption in all complexes of the respiratory chain through the targeted use of substrates, uncouplers and inhibitors [18]. Two milliliters of PBMCs diluted in Mir05 [4 × 10^6^cells/ml Mir05; pH = 7.4; 37 °C] were added to each chamber of the Oxygraph-2 k and equilibrated (endogenous respiration, spin speed 750 rpm). The mitochondrial respiration medium (Miro05) developed by Oroboros contained ethylene glycol tetraacetic acid (EGTA) (0.5 mM), magnesium dichloride (3 mM), lactobionic acid (60 mM), taurine (20 mM), potassium dihydrogenphosphate (10 mM), 4-(2-hydroxyethyl)-1-piper-azineethanesulfonic acid, (HEPES) (20 mM), sucrose (110 mM), and essential fatty acid free bovine serum albumin (1 g/l).

First, 1-µg digitonin (8.1 mM in DMSO) per 10^6^ cells was added for 15 min to permeabilize the plasma membrane of the cells, leaving the outer and inner membranes intact. The capacity of oxidative phosphorylation was determined using complex I-related substrates (CI) glutamate (10 mM; stock: 2 M in H_2_0), malate (2 mM; stock 800 mM in H_2_0), and ADP (2 mM; stock 500 mM in H_2_0) followed by the addition of succinate (10 nM; stock: 1 M in H_2_0). After the addition of glutamate/malate, leak respiration corresponded to state 4 respiration, while the addition of ADP induced state 3 respiration. The stepwise injection of carbonyl cyanide-4-(trifluoromethoxy) phenylhydrazone (FCCP; stock 1 mM in EtOH) uncouples the respiratory chain (ETS) (up to 2.5 µM) and results in degradation of MMP (mitochondrial membrane potential). To measure state 2 leak respiration, oligomycin was added and complex II respiration monitored after the addition of rotenone (0.5 µM; stock 4 mg/mL in EtOH) to each chamber. Residual oxygen consumption, which is oxygen consumption caused by enzymes outside the electron transfer system, was determined after inhibition of complex III via the addition of antimycin A (2.5 µM; stock 5 mM in EtOH) and was subtracted from all respiratory parameters. COX activity (CIV) was measured after ROX determination by applying 0.5 mM tetramethyl-p-phenylenediamine (TMPD; stock 200 mM in H_2_0 + 10 mM ascorbate) as an artificial substrate of complex IV and 2 mM ascorbate to keep TMPD in a reduced state. The autoxidation rate was determined after the addition of sodium azide (**≥ **100 nM; stock 4 M in H_2_0), and COX respiration was corrected for autoxidation [9].

### Citrate synthase (CS) activity

A subsample of 500 µl (2 × 10^6^ cells) of the PBMCs remaining after respiratory measurement was immediately frozen in liquid nitrogen and stored at − 80. After collection, the samples were thawed and citrate synthase (CS) activity was determined photometrically. The reaction medium containing 0.1-mM 5,5’-dithio-bis-(2-nitrobenzoic acid) (DTNB), 0.5-mM oxaloacetate, 50-µM EDTA, 0.31-mM acetyl coenzyme A, 5-mM triethanolamine hydrochloride, and 0.1-M Tris–HCl was mixed and preheated in a water bath for 5 min at 30 °C. Subsequently, 200 µl of cell solution was added to it and CS activity was assessed spectrophotometrically at 412 nm. Measurements were performed in triplicate (2). CS activity was normalized to IU per 1 × 10^6^ cells/ml.

### Gene expression analysis (realtime qRT-PCR)

Total RNA was isolated from 500,000 cells using the RNeasy Mini Kit (Qiagen, Hilden, Germany) in accordance with the manufacturer’s instructions. The RNA Protect (Qiagen, Hilden, Germany) was used to stabilize the cells. A Nanodrop™ 2000c spectrometer (Thermo Fisher Scientific, Waltham, MA, USA) was used to quantify RNA by measuring absorbance at 260 and 280 nm. RNA purity was assessed using the ratio of absorbance 260/280 nm and 260/230 nm. RNA was considered as pure if the A260/A280 ratio was between 1.8 and 2.1. To remove residual genomic DNA, samples were treated with a TURBO DNA-free™ kit in accordance with the manufacturer’s instructions (Thermo Fisher Scientific, Waltham, MA, USA). Complementary DNA was synthesized from 1-μg RNA using the iScript cDNA Synthesis Kit (BioRad, Munich, Germany) according to the manufacturer’s instructions and then kept at − 80 °C. The qRT-pcr was conducted using the CFX 96 ConnectTM system (BioRad, Munich Germany). The Oligonucleotide primer sequences, primer concentrations, and product sizes are listed in Table 3. All primers came from Biomol (Hamburg, Germany), and we used the well-established SYBR Green based assay. In line with MIQUE guidelines, we completed a melt curve for every listed primer pair, whereby each pair produced one significant melt peak. Furthermore, we determined the efficiency of the assay for each primer pair, which ranged from 90 to 110%, and used the optimized concentration of each primer pair. Afterwards, all PCR products were checked using agarose gel electrophoresis to ensure that the right product was amplified. The cDNA for qRT-pcr was diluted 1:10 with Rnase free water (Qiagen, Hilden, Germany) and all samples were analyzed in triplicate. PCR cycling conditions foresaw initial denaturation at 95 °C for 3 min, followed by 45 cycles of 95 °C for 10 s, 58 °C for 30 s, and 72 °C for 29 s. The BioRad CFX manager software was used to analyze gene expression according to the –(2∆∆Cq) method, and expression levels were normalized to those of GAPDH, beta-actin and PGK1.

**Table 3:** Oligonucleotide primer sequences, product sizes and primer concentrations for quantitative real-time pcr; bp: base pairs, conc. concentration

Primer	Sequence	Product size (bp)	Conc. (µM)
ACTB	5′-ggacttcgagcaagagatgg-3′ 5′-agcactgtgttggcgtacag-3′	234	200
SQSTM1	5′-cacctgctgagggcttctc-3′ 5′-agtttcctggtggacccatt-3′	96	200
CI	5′acctgtaaggaccgagaga-3′ 5′-gcaccacaaacacatcaaaa-3′	213	200
CIV	5′-ctgttccattcgctgctatt-3′ 5′-gcgaacagcactagcaaaat-3′	152	200
MAP	5′-agcagcatccaaccaaaatc-3′ 5′-ctgtgtccgttcaccaacag-3′	187	100
CAT	5′-acttctggagcctacgtcct-3′ 5′-cgcatcttcaacagaaaggt-3′	200	150
GPx	5′-gcttccagaccattgacatc-3′ 5′-gtgttcctccctcgtaggtt-3′	170	400
TFAM	5′-tcccccttcagttttgtgta-3′ 5′-atcaggaagttccctccaac-3′	189	400
SOD2	5′-acagcgcatactctgtgtga-3′ 5′-gggggaacaactcaactttt-3′	183	100
PGK1	5′-ctgtgggggtatttgaatgg-3′ 5′-cttccaggagctccaaa-3′	198	200
HSPA	5′-tggactgttcttcactcttggc-3′ 5′-tccggagagttctgggattgta-3′	204	100
GAPDH	5′-ctttgccaacttccttctgc-3′ 5′-ttgattttggagggatctcg-3′	238	200

*PGK1* phosphoglycerate kinase 1, *GAPDH* glyceraldehyde-3-phosphate dehydrogenase, *ACTB* beta-actin, *CI* complex I, *CIV* complex IV, *CS* citrate synthase C, *TFAM* transcription factor A, *CAT* catalase, *GPx* glutathione peroxidase, *SOD* superoxide dismutase, *HSP* heat shock protein, *SQS* ubiquitin-binding protein p62, *MAP-LC3* microtubule-associated protein 1 (*MAP1*) light chain 3 (*LC3*)

### Determination of insulin-like growth factor 1 (IGF-1) in plasma

Based on a protocol previously described by Blum and Breier [19], a well-qualified technician determined IGF-1 plasma levels using an established IGF-binding protein-blocked radioimmuno assay (RIA) at the Children’s Hospital of Giessen. All samples were measured in duplicate in a single assay to avoid inter assay variation.

### MR protocol

MR examinations were performed using a 3 T scanner (Magnetom Prisma Siemens Healthineers, Erlangen, Germany) equipped with a double tuned ^31^P/^1^H volume head coil (Rapid Biomedical, Rimpar, Germany). In addition to localizer scans the protocol included:

- 3D T1w MRI for segmentation (MPRAGE)
- B1 mapping as described in [9, 20]
- 2D ^1^H MRSI at 40 ms TE (sLASER, 12 mm slice, FOV 240 × 240 mm^2^, matrix 20 × 20)
- 3D ^31^P MRSI (FID-CSI, FOV 240 × 240 × 200 mm.^3^, matrix 8 × 8 × 8)
- 2D ^1^H MRSI for detection of water (FID-CSI, 25 mm slice, FOV 240 × 240, matrix 16 × 16, 2° flip angle, 0.54 s repetition time)

Spectra were screened to ensure they fulfilled quality criteria defined by the absence of artifacts and excessive linewidth broadening (> 4 Hz). Volunteer data remaining for final analysis were split into the following groups:

- ^1^H-MRSI: 30 young females, 27 older females, 30 young males, and 29 older males
- ^31^P-HMRSI: 29 young females, 24 older females, 28 young males, and 2 older males

### MR data processing

The program LCModel [21] was used to analyze ^1^H MRSI data, with ^1^H detectable metabolite concentrations being quantified in mmol/l, as described in [9]. It should be noted that the quantification refers to tissue water. The ^31^P MRSI data were analyzed using jMRUI and quantified based on a calibration curve obtained from a dedicated phantom experiment. Special emphasis was put on correcting for differences in coil loadings, as this is important when focusing on gender differences. A detailed description of the procedure will be the subject of another publication. With regard to ^1^H data, the concentration per voxel of ^31^P MR detectable metabolites was divided by the tissue water content obtained from the gray matter (GM) and white matter (WM) fractions to provide final mmol concentrations in tissue water (from ^1^H MRSI data). The tissue fractions were adjusted for the point spread function (PSF) of ^31^P MRSI.

**Fig. 2:**
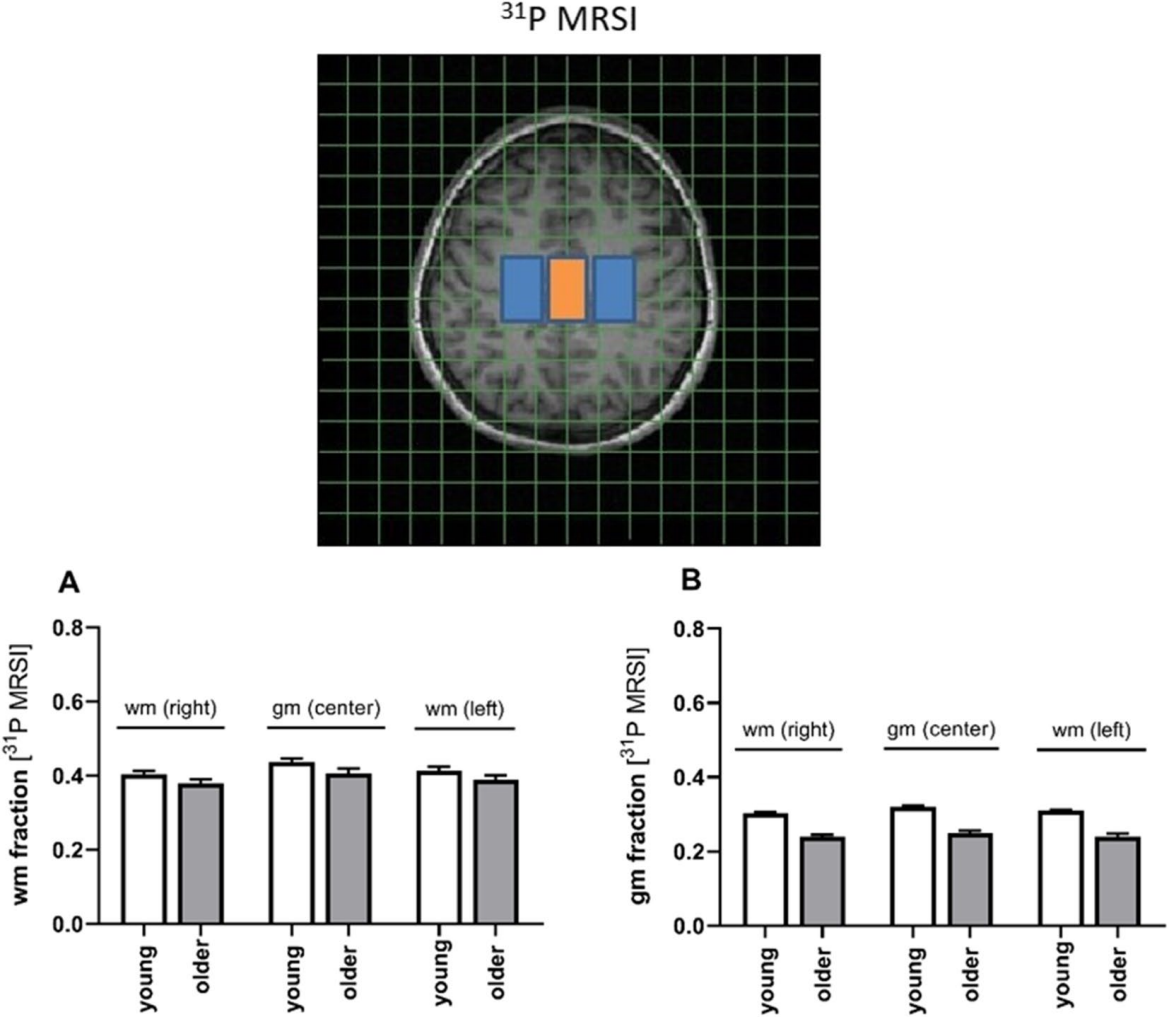
ROIs and tissue fractions for P MRSI (**A**, **B**). The GM region is marked in orange. GM and WM fractions are calculated from segmented tissue imagers filtered using the relevant point spread function (PSF). Note the coarse grid size for P MRSI. In combination with poor PSF, this hampered discrimination between GM and WM, based on the position of the ROI

Data were taken from the target regions as shown in Fig. 2. The graphs in the lower row show the WM and GM fraction from the respective region. While differences were clearly visible for 1H MRSI (data not shown), the poor point spread function for 31P MRSI levels these differences, thus data do not allow for discrimination between GM and WM as shown in the lower panel of the figure. Consequently, all comparisons were performed on averages over all regions.

The metabolites total NAA (tNAA, representing N-acetylaspatate and N-acetylaspartate-glutame) and total creatine (tCr, representing creatine and phosphocreatine) from ^1^H MRSI as well as ATP and phosphocreatine from ^31^P MRSI were included in the analysis. ROI, grid size, and tissue fraction from ^31^P MRSI unphosphorylated creatine (Cre) was obtained by calculating the difference between tCre and PCr.

### Chemicals

Chemicals were of the highest available purity and purchased from Sigma (St Louis, MO, USA) or Merck (Darmstadt, Germany) unless otherwise stated. The aqueous solutions were prepared using deionized, filtered water (Millipore, Billerica, MA, USA).

### Statistics

Values are presented as mean ± standard error of the mean (SEM). Since our previous study revealed significant differences in the measured parameters between males and females [9], we first tested the influence of sex on both, cerebral and peripheral markers of energy metabolism using the two-way ANOVA (Prism 8, GraphPad Software, San Diego, CA, USA). The statistical analysis did not reveal any gender-specific differences in the measured parameters. Therefore, women and men were combined into an old and a young group to increase the significance of the results. Subsequently, group differences were calculated using an unpaired *t*-test (Prism 8, GraphPad Software, San Diego, CA, USA). Statistical significance was defined for *p* values p* < 0.05, p** < 0.01, p*** < 0.001, and p**** < 0.0001. Correlation analysis was performed using linear regression analysis.

## Results

This study was designed to investigate age-associated differences in mitochondrial function in PBMCs and cerebral energy metabolites in healthy young and healthy older volunteers. In addition, we were interested in a potential association between mitochondrial function determined in PBMC and markers of cerebral energy metabolism measured in vivo. We therefore analyzed mitochondrial parameters including ATP levels, citrate synthase activity, and activity of respiratory chain complexes in isolated PBMCs. Cerebral energy metabolites were quantified using ^1^H- and ^31^P-MRSI.

### Subject characteristics and blood data

Some age- and sex-associated differences existed in the PBMCs The ratio of lymphocytes to monocytes was correspondingly lower in older males. However, no more significant differences in PBMC related parameters were observed (Table 2).

### ATP-levels and CIV respiration decrease in PBMCs in aged participants

**Fig. 3:**
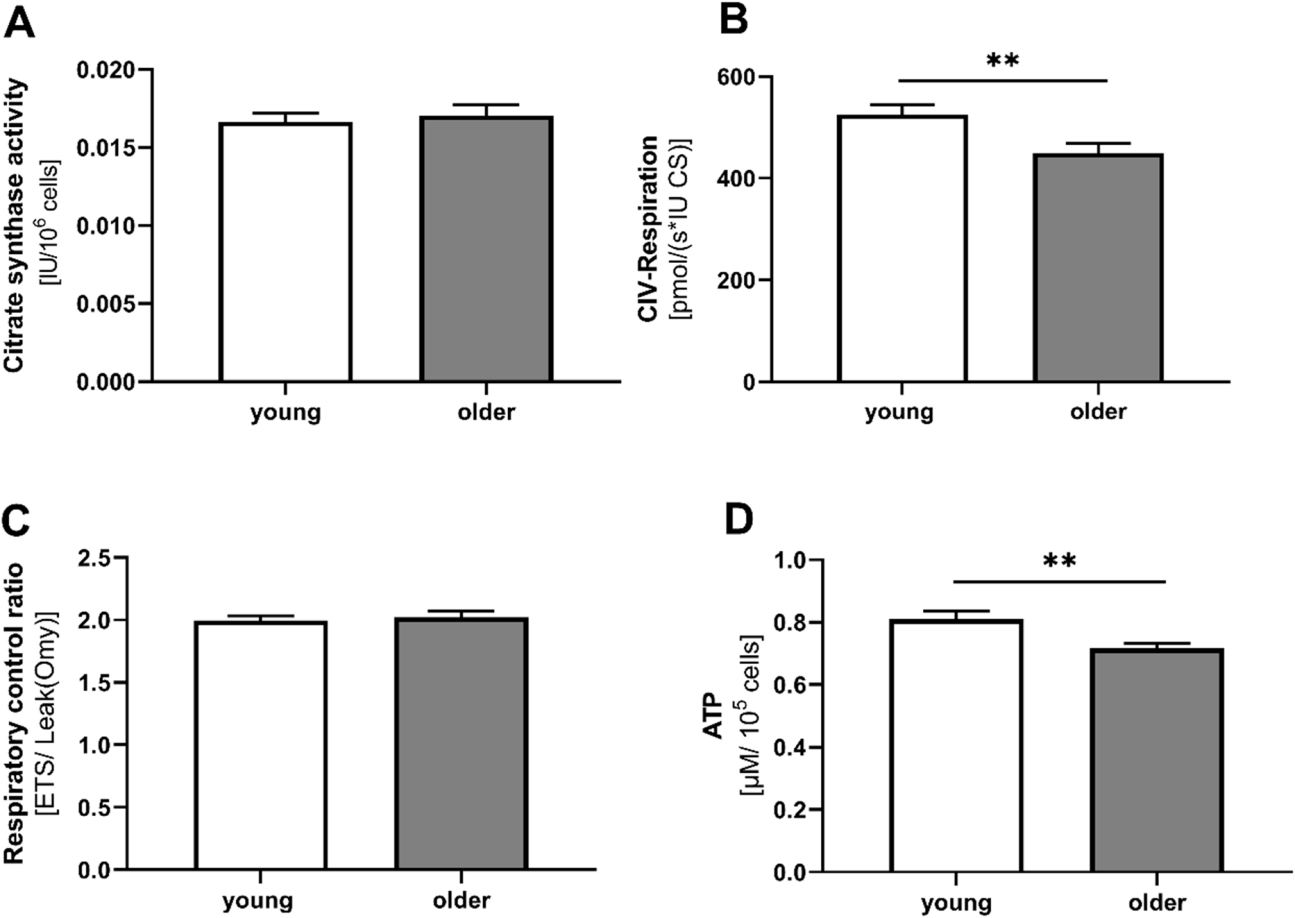
Age-associated differences in mitochondrial function in PBMCs. **A** Activity of the mitochondrial mass marker citrate synthase (CS). The activity of CS did not differ between young and older participants. The figure shows mean values ± SEM; *n* = 58; unpaired *t*-test. **B** Mitochondrial respiration of isolated PBMCs in complex IV of young and older participants measured using high-resolution respirometry. The values are standardized to international units (IU) of citrate synthase activity. Mean values ± SEM; *n* = 58; *t*-test with **p < 0.01. **C** Respiratory control ratio calculated from respiration of ETS/leak after addition of oligomycin in female and male, young and older subjects. The figure shows mean values ± SEM; *n* = 115; unpaired *t*-test. **D** Normalized ATP levels. The figure shows mean ± SEM, *n* = 58, unpaired *t*-test (**p < 0.01). *ATP*, adenosine diphosphate; *ADP*, adenosine triphosphate; *ETS*, electron transport system; *FCCP*, carbonyl cyanide-4-(trifluoromethoxy) phenylhydrazone; *G*, glutamate, *M*, malate; *Omy*, oligomycin; *OXPHOS*, oxidative phosphorylation; *PBMC*, peripheral blood mononuclear cells; *RCR*, respiratory control ratio; *TMPD*, tetramethylphenylendiamine

The key biological function of mitochondria in energy conversion is oxidative phosphorylation (OXPHOS), as performed by the four multiprotein complexes (complexes I to IV (CI–CIV)) of the electron transport chain (ETC), and F0F1-ATP synthase (CV) [7]. The composition of OXPHOS complexes is controlled by both nuclear and mitochondrial DNA and involves certain assembly factors [22] Mitochondrial complexes CI–CIV generate a proton gradient at the inner mitochondrial membrane, and this makes CV produce adenosine triphosphate (ATP) as an energy equivalent [23]. Lack of ATP production is a characteristic of mitochondrial dysfunction [23, 24]. CS activity represents a valid marker for mitochondrial mass [8] and was similar in all investigated groups (Fig. 3A). The activity of complex C-IV was reduced in elderly participants (Fig. 3B). This age-related decline in complex C-IV activity may have led to impaired ATP production, as shown in Fig. 3D. The respiratory control rate (RCR), which reflects the ability to use the proton gradient of the mitochondrial respiratory chain to produce ATP at complex C-V, was not different in PBMC isolated from older participants (Fig. 3C). Apart from complex C-IV, which was lower in older participants, the complex activities of mitochondrial respiratory chain complexes normalized to CS activity were similar in all age groups (see Table 4).

**Table 4:** Mitochondrial complex activity

Group	Young	Older	*p*-value
Endogenous respiration	137.7 ± 4.2	130 ± 5.1	0.25
OXPHOS	384.9 ± 15.2	370.6 ± 12.3	0.46
CII_ETS_	276.7 ± 10.8	262.5 ± 9.6	0.33
CI	133.6 ± 5.5	126.9 ± 5.7	0.40

Mean ± SEM, *n* = 58; *t*-test with *p < 0.05. *CI* complex I, *CII* complex II, *ETS* electron transport system, *OXPHOS* oxidative phosphorylation, *RCR* respiratory control ratio, *Omy* oligomycin

### Age-associated differences in gene expression patterns of PBMCs

Gene expression analysis was conducted in PBMCs taken from young and older participants. No significant differences were observed when gene expression was compared between young and older participants (see Table 5).

**Table 5:** Gene expression

Participants			*p*-value
Group	Young *n* = 52	Older *n* = 58	
C-I	325.9 ± 76.2	221.9 ± 24.5	0.18
C-IV	74.1 ± 76.6	68.8 ± 5.1	0.53
CS	168.0 ± 17.1	204.9 ± 18.1	0.14
TFAM	65.8 ± 10.6	50.5. ± 3.6	0.15
CAT	170.8 ± 12.7	205.5 ± 17.4	0.12
GPx	265.2 ± 21.4	259.3 ± 18.0	0.83
SOD	355.3 ± 42.5	334.8 ± 21.6	0.66
HSP	470.3 ± 35.2	479.6 ± 32.1	0.84
SQSTM1	423.0 ± 34.3	456.2 ± 29.3	0.46
MAP	250.0 ± 38.4	206.2 ± 20.3	0.31

Relative gene expression in PBMCs. *n* = 56; Mean ± SEM, t-test against old participants. *CI* complex I, *CIV* complex IV, *CS* citrate synthase C, *TFAM* transcription factor A, *CAT* catalase, *GPx* glutathione peroxidase, *SOD* superoxide dismutase, *HSP* heat shock protein, *SQS* ubiquitin-binding protein p62, *MAP-LC3* microtubule-associated protein 1 (*MAP1*) light chain 3 (*LC3*). Results are normalized to the expression levels of GAPDH glyceraldehyde-3-phosphate dehydrogenase, beta-actin and PGK1 phosphoglycerate kinase 1

### Insulin-like growth factor (IGF-1) plasma levels decrease during aging

**Fig. 4:**
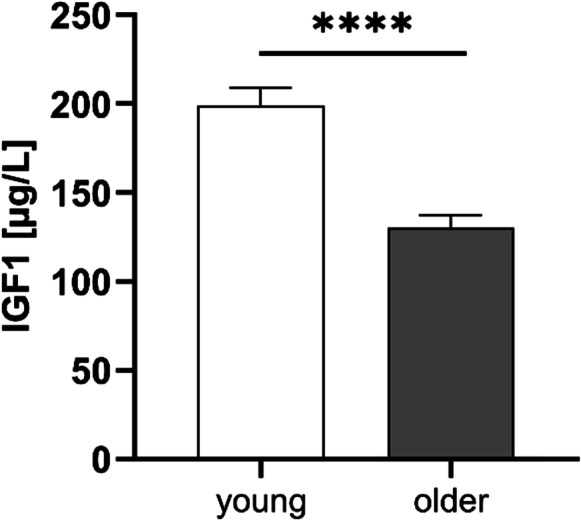
Age-associated differences in plasma levels of insulin-like growth factor 1 (IGF-1). *t*-test was performed; *p*-values are defined as (*p < 0.05; **p < 0.01, ***p < 0.001, ****p < 0.0001)

The growth hormone (GH)-IGF-1 system is part of the somatotropic hypothalamic-pituitary axis and is involved in the regulation of metabolism, somatic growth, and aging. The effects of GH/IGF-1 on aging mitochondria have recently been reported, indicating that IGF-1 may contribute towards mitochondrial dysfunction in older persons [25]. IGF-1 levels were determined in plasma samples and were found to be significantly lower in older participants (Fig. 4).

### Cerebral energy metabolites change during aging but ATP brain levels remain unaffected

**Fig. 5:**
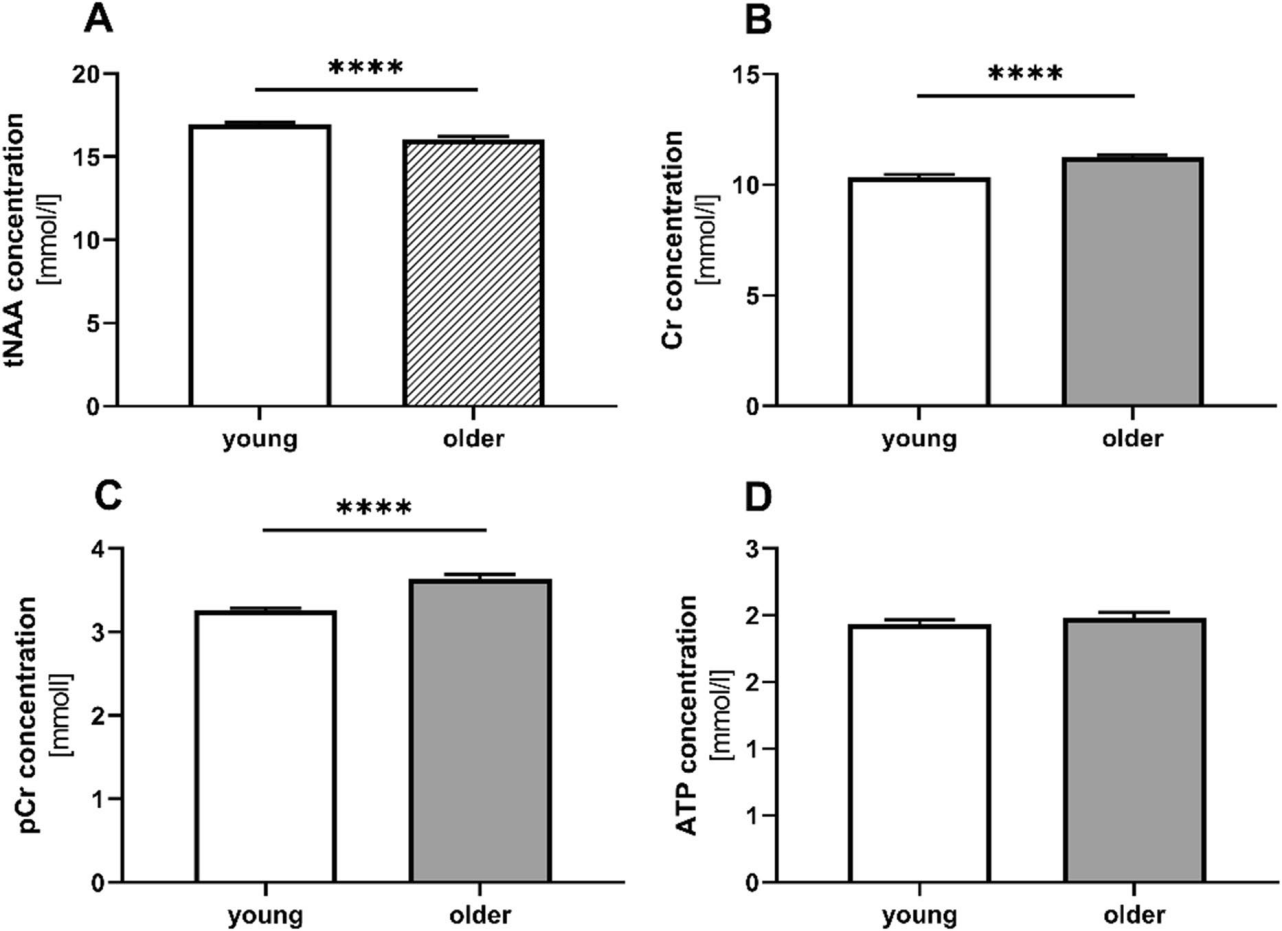
Age-associated differences in cerebral parameters. Total N-aspartyl-aspartate (NAA) (**A**), total creatine (Cr) (**B**), phosphocreatine (pCr) (**C**) and total ATP levels (**D**) in the brains of young and older female and male subjects**.** Concentrations are given in millimoles divided by tissue water volume. Data represent means ± SEM; **A** and **B** ^1^H data young: *n* = 59, older *n* = 56; **C** and **D** from.^31^P data; *t*-test was used to calculate significance for each metabolite (*p < 0.05, **p < 0.01,***p < 0.001,****p < 0.0001)

Due to poor PSFs for the ^31^P-MRS data, concentration differences in PCr and ATP were calculated by averaging the results for all three regions shown in Fig. 1. These results thus represented concentrations averaged over WM and GM, whereby the fractions can be found in the last row of Fig. 1. The three most prominent signals in ^1^H-MRSI data represent metabolite group totals for the respective compounds. Totals are indicated by a t in front of the abbreviation. In terms of energy metabolism, Fig. 4 shows the results for the four metabolites. The NAA concentration was significantly reduced in the brains of older participants (Fig. 5A). Older participants had significantly higher total creatine and phosphocreatine levels than the young controls (Fig. 5B, C). Cerebral ATP levels were similar in all investigated groups (Fig. 5D).

### ATP blood levels do not correlate with cerebral ATP levels or energy metabolites

**Fig. 6:**
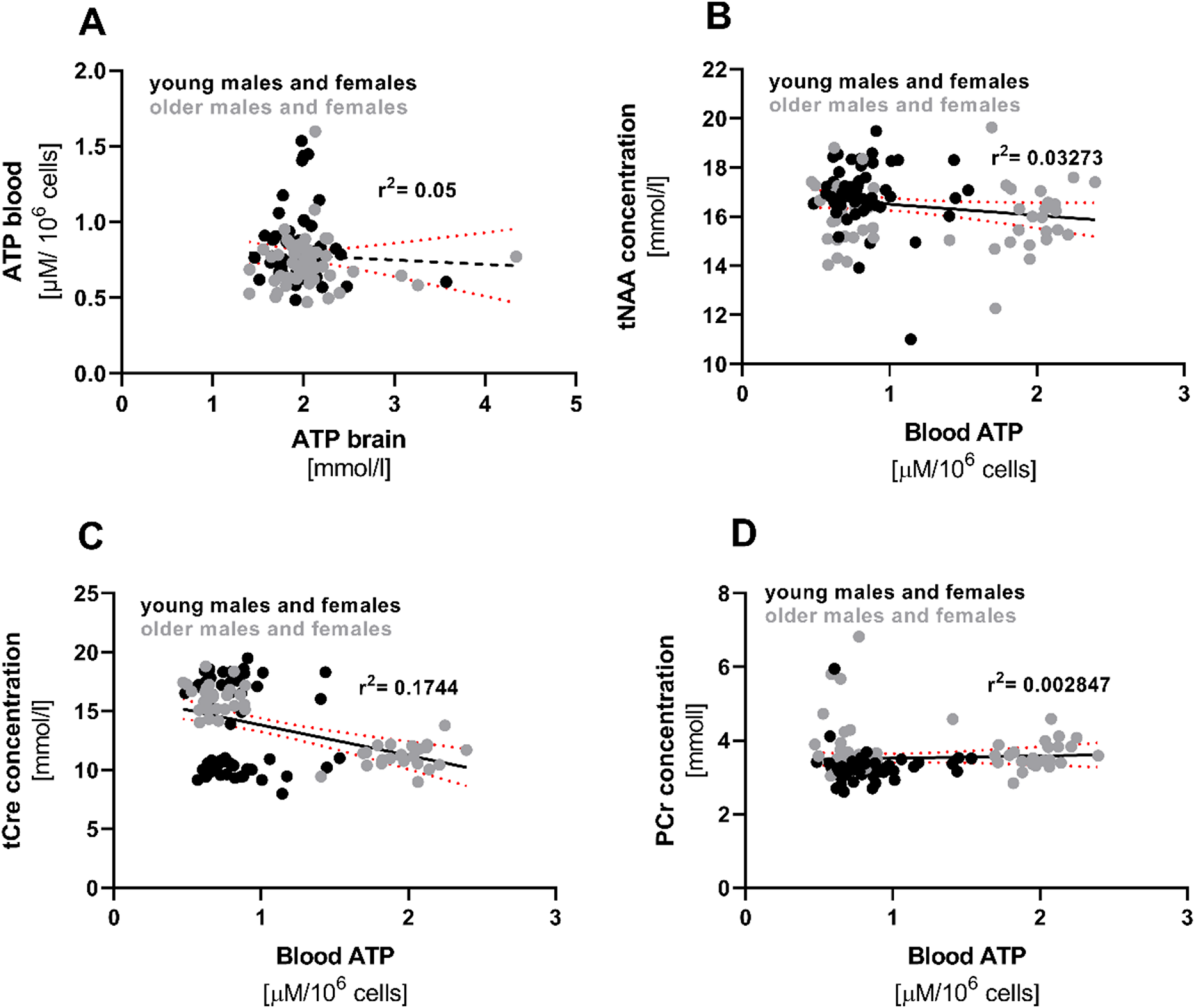
Linear regression analysis was performed to analyze the correlation between levels in young and older participants of ATP in blood and brain (**A**), cerebral energy metabolites tNAA (**B**), Cr (**C**) and PCr (**D**)

Levels of ATP in PBMCs (ATP blood) did not correlate either with cerebral ATP levels (ATP brain) (see Fig. 6A), or with the cerebral energy metabolites tNAA, tCr, or PCr (Fig. 6B–D).

## Discussion

Our cross-sectional observational study investigated mitochondrial function in PBMCs and energy metabolites in the brains of young and older healthy participants and determined associated gene expression in the levels of PBMC and IGF-1 in plasma samples. The activity of mitochondrial respiratory chain complex IV (CIV) and ATP levels were reduced in PBMCs isolated from older participants. Respiratory control ratio and citrate synthase (CS) activity were unchanged. Serum levels of IGF-1 were significantly reduced in older participants. Expression of genes involved in mitochondrial activity, antioxidative mechanisms, and autophagy were unaffected by age. In brains of older participants, tNAA levels were lower and tCr and PCr levels were higher. Cerebral ATP levels were similar in young and older volunteers. Energy metabolites in PBMC were not correlated with those measured in the brain.

### Mitochondrial function and gene expression in PBMCs

During oxidative phosphorylation, mitochondrial respiratory chain complexes generate a proton gradient at the inner mitochondrial membrane, which stimulates the production of ATP [7]. Since CS, which is a marker of mitochondrial mass, was unaltered [8] the decrease in ATP levels was mainly attributable to the reduced activity of complex IV in the PBMCs isolated from older participants.

Bektas et al. investigated age-associated changes in human CD4 + T lymphocytes isolated from PBMCs [26]. In line with our CS data, the number of visualized mitochondria in CD4 + T lymphocytes using electron microscopy was similar in older and young participants. Reflecting the decrease in CIV activity in our study, mitochondrial respiration was impaired in CD4 + T lymphocytes from older subjects. Interestingly, mitochondrial proteins, including CIV proteins, involved in the electron transport chain, were overrepresented in the cells of older participants. However, CD4 + T-lymphocytes showed dysregulation within oxidative phosphorylation complexes and energy metabolism pathways [26].

Endogenous respiration was unchanged in PBMCs isolated from older donors, confirming the findings of Yabro et al., who, using a Seahorse XFp system, observed that aging did not impair the rate of LPS-induced oxygen consumption during glucose deprivation in monocytes isolated form older donors [27]. In a recent study, Herpich et al. investigated mitochondrial function in PBMCs isolated from older patients with fatigue syndrome and older matched control persons [28]. Routine and maximal mitochondrial respirations were significantly reduced in PBMCs isolated from the older patients. Since no young controls were assessed, age-related conclusions could not be drawn from that study [28]. We showed reduced CIV activity in the PBMCs of older participants, which is partly in agreement with a recent study showing reduced CS activity, reduced endogenous respiration, and lower activity in the respiratory chain complexes, including CIV, of PBMCs isolated from young male, as compared to young female donors [9]. However, the latter study included only nine male and 15 female subjects, so the results may not be robust. The following table summarizes relevant studies of mitochondrial function in PBMCs during aging and in different disease models (Table 6).

**Table 6:** Further studies in PBMCs focused on mitochondrial bioenergetics during aging and in different disease patterns; *n.d.* not determined. (↑ increase, ↓ decrese, (↓); (↑) tends to decrease/increase)

Mitochondrial function in PBMCs	Subjects	ATP levels	Mitochondrial respiration	Literature
Current study	Young and older males and females	↓ older participants	(↓) older participants	
Herpich et al. (2021)	Geriatric patients with and without fatigue syndrom	↓ geriatric patients	↓ geriatric patients	[28]
Gumpp et al. (2021)	Females (18–60 years) with depressive disorder	n.d	↔ between depressive and non-depress women	[29]
Sriwichaiin et al. (2023)	Middle-aged (< 65 years) and older adults (> 65 years)	(↓) older adults	↓ older adults	[30]
Gangcuanco et al. (2020)	> 40 years old living with HIV	n.d	↓ CI protein levels	[31]
Apaijai et al	Older adults (> 70 years) with and without MCI	↓ MCI patients	↓ mitochondrial proton leak in MCI patients	[32]

### IGF-1 blood levels

The GH-IGF-1 signaling pathway is known to be affected by the aging process and plays a major role in neuronal development processes [33]. The expression of the neurotrophic factor IGF-1 is particularly high in the central nervous system and reaches a maximum during puberty. With aging, central IGF-1 declines by up to 30% and in the periphery by up to 70% [34]. The multifaceted influence of IGF-1, both in the maintenance of cognitive abilities and in the cellular domain, suggests that it protects neurons from oxidative stress. However, this effect has not yet been conclusively understood [35]. As the IGF-1 receptor is also expressed in PBMCs, these peripheral cells may also be affected by IGF-1 [36]. In addition, it appears possible that IGF-1 influences mitochondria in many different ways [37]. It has been shown that in the hippocampus of IGF-1-deficient mice, OXPHOS, and ATP levels are lower and ROS levels higher than in older mice with normal IGF-1 levels [38]. Moreover, the authors reported that IGF-1 had an impact on mitochondrial biogenesis via activation of the transcription factor PGC1-α, and that it regulated mitochondrial apoptosis via the phosphatidylinositol 3-kinase forkhead-box-protein (PI3K/FOXO) pathway [38, 39]. For example, low-circulating IGF-1 levels seemed to correlate with reduced cognitive performance. In contrast, long-lived dwarf mice that were deficient in GH and IGF-1 were observed to have a prolonged lifespan and normal cognitive functions [40]. In our study, we found significantly reduced IGF-1 levels in the plasma of older participants, as was the case in several other studies involving human and animal models of aging [39-41]. Based on these data, we hypothesize that reduced IGF-1 levels in older participants may be related to reduce CIV activity and lower ATP levels in PBMCs.

### Cerebral energy metabolites

Magnetic resonance spectroscopic imaging was used to determine metabolites which may be considered as markers of energy metabolism tNAA and tCr (obtained from ^1^H-MRSI) and the high-energy metabolites tNAA, Cre, PCr, and ATP in the brains of young and older participants. The tNAA signal orginates from the amino acid NAA and the peptide N-acetylaspartate-glutamate and is considered to be a marker of mitochondrial function and neuronal health that reaches concentrations over 10 mM in the brain [42, 43]. We observed reduced total NAA and enhanced overall concentration of Cr and PCr in older subjects, which is in agreement with the literature. Systematic reviews of brain metabolite changes, as measured using ^1^H-MRS in healthy persons, have also concluded that levels of tNAA may decrease, and of Cr increase, in the brains of humans as they age [44-46]. A whole-brain MRSI study investigating brain metabolic changes under normal aging by collecting data during every decade of the lives of healthy subjects from 20–70 years of age found that healthy aging is associated with region-dependent alterations in brain metabolism, including a decrease in NAA [47]. A study in rats has shown that tNAA is synthesized in brain mitochondria [48]. MRSI data from patients with mitochondrial disorders and patients suffering from multiple traumatic mild brain injury have further indicated that NAA may be influenced by mitochondrial function in the brain [49, 50]. It has therefore been assumed that the decrease in tNAA was associated with mitochondrial dysfunction [51]. More recent investigations that used ^13^C/^1^H-MRS to quantify tricarboxylic acid cycle rates in the brains of young and older volunteers found that mitochondrial metabolism in elderly subjects was approximately 30% lower. On an individual level, the reduction correlated strongly with lower NAA concentrations, which is consistent with chronic reductions in brain mitochondrial function [52]. However, ^31^P-MRS-based determination of PCr- and ATP-concentrations, which are considered to directly reflect total high-energy phosphate turnover [53], revealed no change in cerebral ATP concentrations. One possible explanation for constant ATP levels in the brains of elderly subjects arises from the balance between PCr and ATP. PCr is an energy-rich reservoir associated with ATP, and when catalyzed by creatine phosphokinase, ATP is formed from PCr and vice versa. The equilibrium state in this reaction favors ATP formation, so that energy demands that exceed the capacity of cells for ATP synthesis are initially met by a shift in this equilibrium, with ATP concentrations being held constant by PCr hydrolysis [53, 54]. Schmitz et al., recording whole brain 31P-MRS without any localization, reported a negative correlation between age and ^31^P-MRSI ATP signals [55]. However, direct comparison of data from young (< 30 years) and older subjects (> 60 years) revealed no significant differences in ATP levels, which agrees with our findings. An earlier study that used phosphorus ^31^P-MRSI to investigate the relationship between brain energy metabolism and healthy aging by assessing tissue-specific differences in metabolites, including ATP [56] reported no age-related increase but a significant increase in PCr, which is confirmed by our data. The tCre levels were significantly enhanced in elderly subjects in our study, which is in line with most published ^1^H-MRSI studies (see [46]). However, in addition to other differences, the previously published studies do not focus on the same brain region and brain matter compared to this one.

### Correlation between mitochondrial function in PBMCs and central energy metabolites

This is the first study to evaluate correlations of levels of energy metabolites in circulating blood cells with those in the brain. Our data show that ATP levels in PBMCs are not correlated with either cerebral ATP levels or any other energy related metabolites in the brain. Although elderly participants had significantly lower levels of ATP in PBMCs, and of NAA in their brains, peripheral and central energy metabolism do not appear to be related. Recently, we assessed the correlation between mitochondrial function in PBMCs and brain energy metabolites in healthy young men and women [9]. No sex-associated differences were found in PBMCs and the brain, and the determined parameters were not significantly correlated. Energy metabolism in the periphery and the brain has mainly been addressed in studies investigating mitochondrial dysfunction in diseases such as myotonic dystrophy, amyotrophic lateral sclerosis, and Huntington’s disease [57, 58]. However, only Van Diemen et al. searched for a direct correlation between central and peripheral energy metabolites, which was in patients with Huntington’s disease. This study used ^31^P-MRS to measure mitochondrial function in the cerebral cortex and the calf muscle. Mitochondrial function was also assessed ex vivo in circulating PMBCs [58]. However, in line with our findings, a correlation between peripheral and central mitochondrial function was not observed. We detected an age-related decrease in ATP in peripheral blood cells but not in the brains of the test persons. Accordingly, our data revealed no correlation between energy metabolites in PBMCs and the brain, which may be because PCr hydrolysis maintains ATP at constant levels in the brains of healthy older subjects [59]. This hypothesis is supported by the significant correlation between cerebral PCr and ATP. Age-related changes in levels of energy metabolites in the brains of the older test persons could be confirmed by the changes we determined in the levels of tNAA, Cre, and PCr.

## Conclusion

Age-related bioenergetic changes have been detected in peripheral blood cells and the brains of healthy elderly people. However, ATP-levels in blood cells did not correlate with energy metabolites in the brain. While ATP levels in PBMCs may be a valid marker for age-related mitochondrial dysfunction in peripheral tissues of humans, the energy buffer PCr appears to maintain cerebral ATP at constant levels.

### Supplementary Information

Below is the link to the electronic supplementary material.

Supplementary file 1 (DOCX 729 KB)

## Data Availability

The dataset generated during this study is available from the corresponding author on reasonable request.
