# Supplementary material for: Age-related changes in energy metabolism in peripheral mononuclear blood cells (PBMCs) and the brains of cognitively healthy seniors
Source: GeroScience. 2023 Jun 13;46(1):981–98. doi: 10.1007/s11357-023-00810-9 (PMC10828287; doi:10.1007/s11357-023-00810-9)

**Supplementary:**

**Fig 1**: Example image and cell types and CD expressions of PBMC FACS analysis.


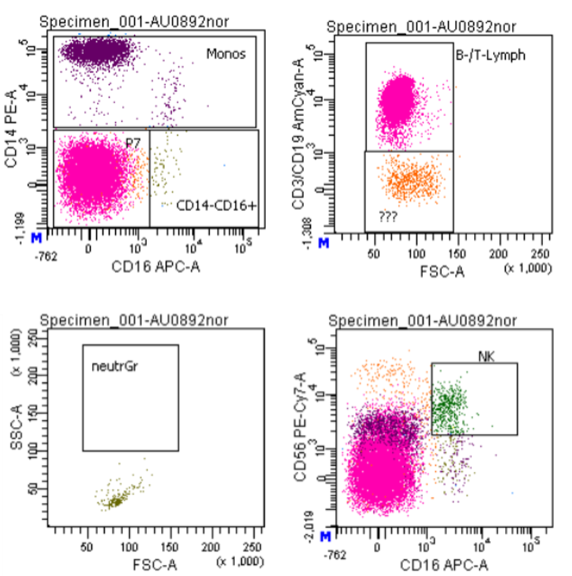


**Tab S1:**

| **Cell type** | **CD-Expression** |
| --- | --- |
| NK-cells | CD56+, CD16+ |
| monocytes | CD14+, CD16- |
| T-lymphocytes and B-lymphocytes | CD14-. CD16-, CD3+, CD19+ |
| neutrophilic granulocytes | CD14+, CD16+ |

NK natural killer cells CD Cluster of differentiation.

**Tab S2:** Results of CERAD test (Z-values ± SEM)

| **Variables** | **z-value ± SEM** |
| --- | --- |
| Semantic fluency (animals) | -0.03 ± 0.13 |
| Boston Naming Test | 0.26 ± 0.12 |
| Wordlist Learning Total | -0.38 ± 0.16 |
| Retrieve word list | -0.23 ± 0.13 |
| Saving Word list | -0.14 ± 0.11 |
| Discriminability | 0.08 ± 0.13 |
| Drawing figures | 0.25 ± 0.14 |
| Retrieve figures | -0.05 ± 0.16 |
| Saving figures | -0.08 ± 0.13 |
| Phonematic fluency (S-words) | 0.79 ± 0.16 |
| Trail Marking Test, Part A | 0.43 ± 0.16 |
| Trail Marking Test, Part B | 0.45 ± 0.15 |
| Trail Marking Test, B/A | 0.07 ± 0.17 |

**Fig S2**: Linear regression analysis was performed to analyse the correlation between blood- and brain ATP levels as well as brain energy metabolites tNAA, Cr, pCr (Fig. A-N).


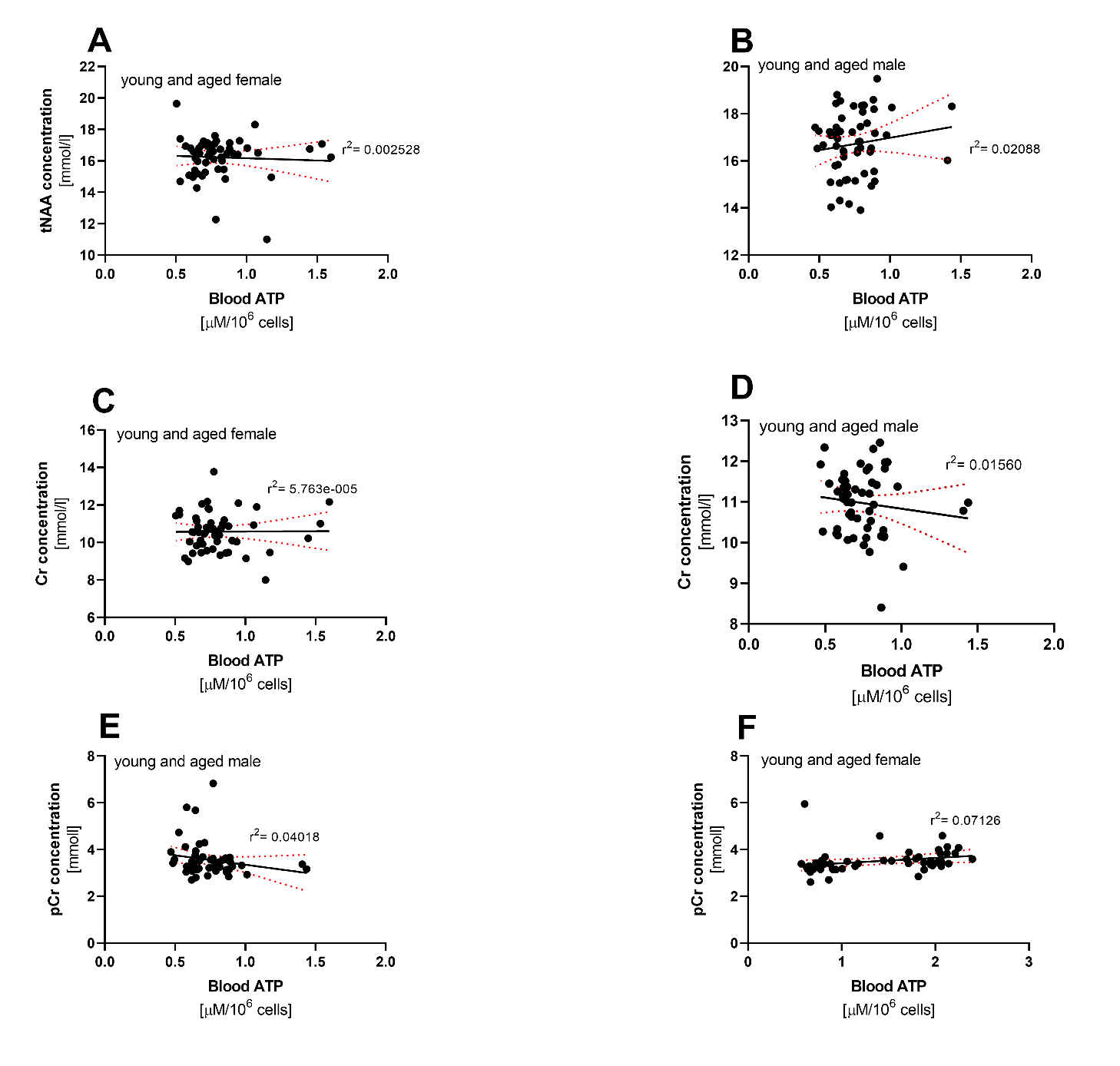


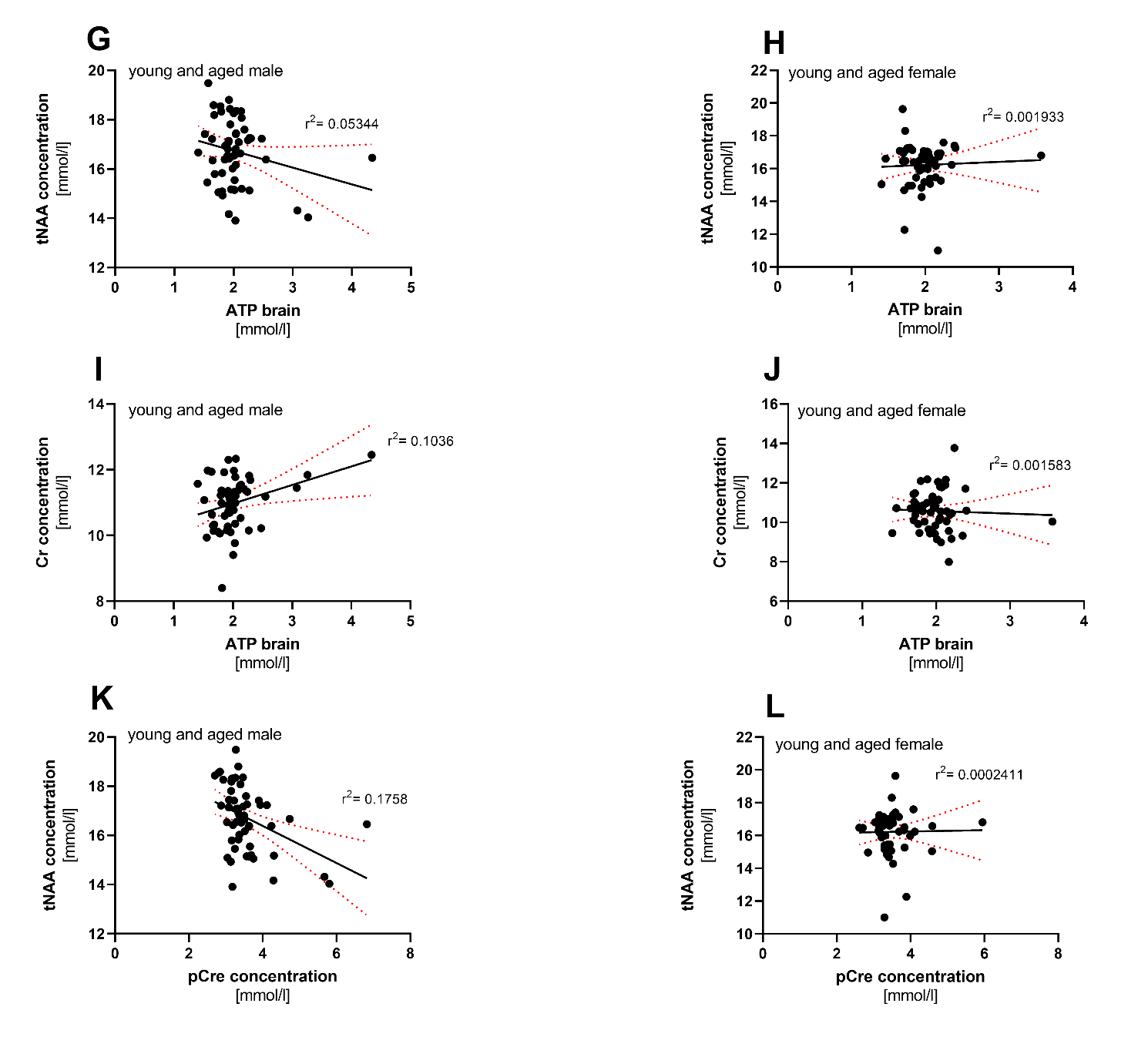


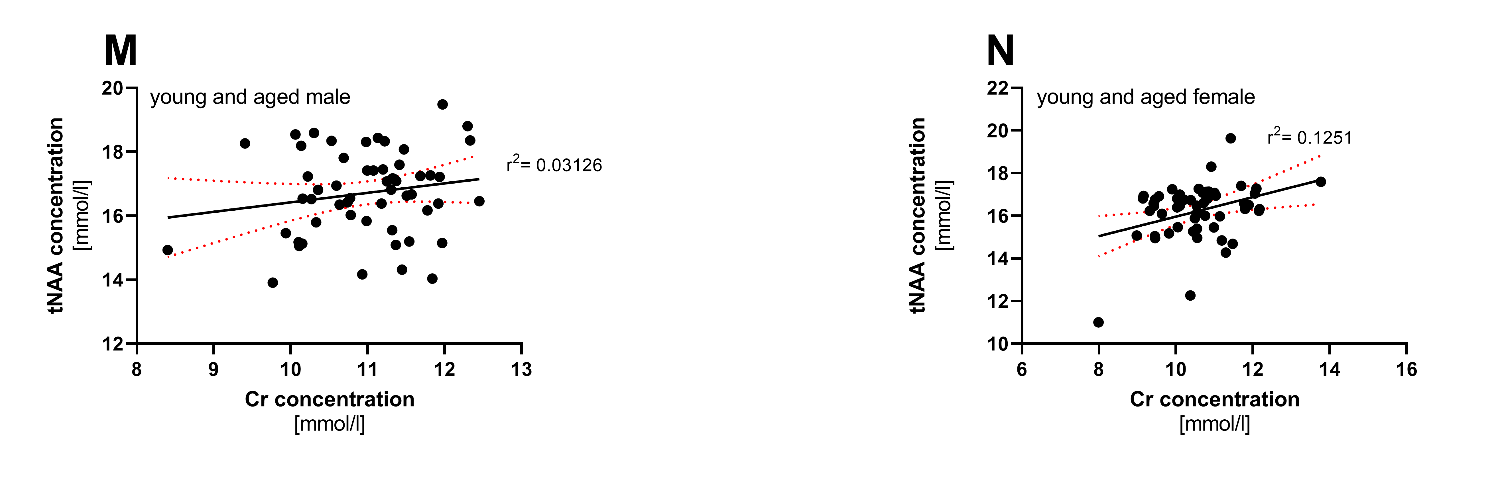

Supplement: Supplementary file 1 — (DOCX 729 KB) [file 11357_2023_810_MOESM1_ESM.docx]
